# The (cognitive) future of motor control and learning

**DOI:** 10.3389/fspor.2023.1181808

**Published:** 2023-09-13

**Authors:** Dirk Koester

**Affiliations:** Faculty Sport Sciences and Personality, Business & Law School, Berlin, Germany

**Keywords:** motor learning, skills, motivation, emotion, representation, coaching

## Abstract

An ongoing debate exists regarding the compatibility of dynamic systems theory (DST) and symbol processing accounts (SPA), where SPA assume abstract representations and processing. Another aspect under discussion is if either one appropriately describes and explains motor control and the modification of motor skills. Both frameworks have their strengths and weaknesses. DST provides mechanistic explanations and takes system complexity and the environment into account without reference to mental entities. System behaviour is described mathematically and considered deterministic. In contrast, SPA propose that abstract content, that is, mental representations of the (own) body, and task requirements are critically important for movement control. It is argued that neither approach nor an (unaccomplished) unification of these frameworks can achieve a comprehensive understanding of motor control and learning. In this perspective article, it is argued that further effective sources of motor learning, such as emotional support and motivational guidance, have the potential to improve and preserve motor skills indirectly and should, thus, be recognised. Qualitative approaches focussing on understanding the athlete and the situation might be appropriate for applied work.

## 1. Introduction

This perspective article calls for terminological precision when referring to theoretical foundations and advocates a broader perspective on motor control and learning. Learning refers to the modification of our action repertoire. More specifically, motor (or skill) learning refers to the long-term process of acquiring and improving (actually changing) goal-oriented movements, specifically muscle activation patterns [cf. (1–3)]. In contrast, motor adaptation can be differentiated from skill learning in that it refers to shorter responses during learning [cf. (4), including a discussion of the role of errors, reinforcement, and reward]. Skill learning can proceed implicitly or explicitly. Thus, explicit (including verbal) input from the environment or other persons, such as observation, instruction, or feedback, can foster motor learning.

A comprehensive understanding of skill learning is essential because motor skills are required throughout the lifetime and in almost all aspects of daily life. Strengthening skill learning is necessary to achieve high performance levels and reduce sedentary behaviours and associated health-related costs for societies. Relatedly, physical activity and motor abilities have declined significantly in (mainly Western) societies in recent decades [e.g., (5–8)], with some recent stagnation (9, 10). A comprehensive perspective is needed not only to maximise peak (sport) performance but also to optimally support the acquisition, improvement (e.g., in physical education), and rehabilitation after impairment of basic motor skills.

Understanding human skill learning is not trivial because of the complexity of the movement system, incl. all bones, joints, and muscles [for related discussions such as the motor–action controversy, see (11)]. Moreover, there are various other influential factors such as external stimuli (physical to social), biological energy supplies, nervous control structures, and psychological states [cf. (12)]. All these factors should be considered to provide optimal support for skill learning. A comprehensive scientific theory may reduce potential influences to the basic effective factors, and such a theory needs to explain a wide range of actions. Not limited to sports, we need to understand how humans perform actions with the highest precision demands (e.g., dart throwing and clockmaking), maximum force (e.g., weightlifting and construction work), high speed (e.g., sprinting and running), or even a combination thereof (e.g., pole vaulting and forging). Given the complexity of human movement control (incl. aims), a comprehensive theoretical basis is required for optimal support.

## 2. Theoretical approaches

Two contrasting classes of scientific approaches, namely, dynamic systems theory (DST) and symbol processing accounts (SPA), will be discussed to understand human movement control and learning. The underlying foundations appear incompatible, and missing factors will be considered, contributing to the difficulty of reaching a comprehensive, unified account.

### 2.1. Dynamic systems theory

To understand (i.e., describe) the overall behavioural variability of highly complex systems (e.g., humans), DST considers all the system components (e.g., body parts, muscles, and organs) and environmental influences. Any system behaviour is construed as a pattern (of change or action), and these patterns can either be stable over a range of relevant variables or change within a small (critical) range of system variables. The interplay of variables and, thus, the system behaviour are described in a set of mathematical formulas. The mathematical equations of DST originate in physics [cf. (13)] but have been applied explicitly to “both animate and inanimate systems” (14, p. 3) [see also (15)]. Movement coordination and cyclic movements have often been focussed on. According to DST, motor control is inherently less demanding (actually, it assumes no control entity) because the properties of the body parts (e.g., length, masses, and tissue stiffness) contribute to movement control [as system subcomponents, e.g., damping of the movement end; cf. (16)].

The main objective is to describe the changes in a system *over time*, which is an important aspect of DST. The term “dynamic” refers specifically to the dependency of variables on their values at an earlier point in time. Technically speaking, the previous system state at time *x_n_*_−1_ determines the system state at time *x_n_* (variables may, of course, also be influenced by other variables). Such mathematically described developments over time indicate causal relationships. That is, the changes in system states are *deterministic* (14, p. 19) [cf. (17) for an application to psychology; (18), as cited in (19)]. Deterministic processes are interesting because they have the potential to (fully) explain phenomena, although such explanations require measuring (all) relevant variables to go beyond a mere description (i.e., to provide evidence for the explanation).

Furthermore, the deterministic nature of DST makes any higher-order control structure unnecessary (14, pp. 26). The changes in a system behaviour (pattern) result mechanistically from the current system state and the previous state. This is referred to as *self-organisation* and a central property of DST (14, p. 8, ch. 2). Consequently, the changes in the system state over time (e.g., the unfolding of movements) are *fully* determined by current and previous variable values. No mental state, symbolic representation, or processing is required for system control [e.g., for movement execution; cf. (20)].

Although rigorous regarding the target phenomenon (deterministic with mathematical precision), DST is interesting because it can describe a wide range of movements with a single set of equations. The system behaviour is described as stable and flexible (14, p. 20). For example, walking (a cyclic movement pattern) is more or less constant (stable) across a speed range. If the speed increases to a certain point, then the movement pattern changes drastically (e.g., into running) within a narrow speed range (flexible). Similarly, for running, the movement pattern is stable across a wider speed range. Furthermore, such models do not contain logical (or intellectual) relations, for example, in the sense of *if* (the speed is greater than *X*) *then* (switch to running)—*rules*.

Based on the DST framework, multiple variable learning episodes (experience) should generalise and improve specific skills (21). These ideas have been *conceptually* applied to practise, in that athletes are given multiple different movements as instructions to improve and maximise their motor skills [e.g., (22–24)]. Proponents *assume* that stable movement patterns (which are optimal for a given situation) emerge due to widely varied experiences.

### 2.2. Symbol processing accounts

In contrast, symbol processing accounts assume that human actions, that is, body movements, are controlled by a motor plan or instruction (25). These commands to move are abstract entities (symbols or internal representations) with specific meanings. The motor commands (and their sequence) result solely from computations (information processing) on smaller units, such as movement parts, intentions, and other external information such as distance to goal. The origin of SPA dates back to the *cognitive turn* of the 1960s, when the “mind and brain as computer” metaphor developed and *plans* as hierarchical descriptions (from strategies to muscle commands) became central to understanding behaviour (26). Plans were computed as sets of instructions for the intended behaviour. These computations can be compared metaphorically to sentence construction [from words and grammar rules (27), but see (28)]. Hence, SPA can be said to be language-like.

In addition to mechanistic motor control such as reflexes, SPA extends the mode of movement control to voluntary actions, which do not depend on any stimulus. This “openness” to mental states (e.g., goals and reflections) is one strength of SPA as it implies a non-deterministic perspective on human actions [i.e., it is not physically causally closed; e.g., (29)]. The openness to mental states provides a theoretical basis for applied work, for example, mental training (30–32). In addition, these accounts can incorporate *reasoned* decisions in computations (e.g., logical thinking or conditional choices); that is, an argument can be incorporated, which appears natural given that many of our actions are perceived as reasoned decisions. Another advantage is that researchers can perform computations (static, statistical models) based on empirical data.

A fundamental issue with SPA is that it remains unknown how representations “emerge” from physiological activity [cf. 33, 34]. In a similar vein, it remains unclear how a representing neuronal signal can *stand for* something else, namely, the entity it refers to, and how mental states (e.g., intentions, goals, arguments, and decisions) influence the biological level, that is, the functions of the nervous system. SPA is described to be disembodied [processing is understood in terms of symbols or propositions, and motor control parameters are largely ignored; cf. (35–37)].

The so-called degree-of-freedom problem is another illustrative limitation of SPA [first described by (38); (39, 40) for discussion]. Given a specific movement goal, for example, reaching an endpoint (e.g., a cup of tea), there are many trajectories to perform the movement. Yet, such a movement does not pose any difficulty in real life. Nevertheless, evaluating all possible trajectories to determine the optimal one seems impossible. The fact that even computers cannot achieve such calculations in real time (as fast as humans or animals perform such actions) indicates that the approach is conceptually inadequate; hence, other research strategies are pursued [e.g., (41)].

## 3. Discussion

The SPA and DST frameworks can be construed as diametrically opposing endpoints of a spectrum of theories, both having advantages and disadvantages. Unfortunately, they cannot be “merged” without losing the advantages; these frameworks are incommensurable paradigms (42, 43). In brief, DST is deterministic and does not need “expert supervision” [i.e., a dedicated control structure, cf. (14, pp. 8, 26; 20)]. In addition, DST makes mathematical predictions for all time points, and for a prediction, (initial) variables need to be known with infinite precision (“exact”). Consequently, DST must be seen as descriptive in nature and not explanatory. DST cannot be used for a specific case because not all variables can be measured nor measured exactly.

In contrast, SPA are open to mental influences and cannot be deterministic (in the sense of physical causation). The SPA *are* applicable to empirical data, that is, to specific cases, mostly as statistical associations (rather than full deterministic relations). Furthermore, SPA are easier to develop, for example, in light of new data or insights; it is easier to edit, add, or remove variables. Because the advantages of one framework are almost the disadvantages of the other (e.g., a theory or framework can either be deterministic or not), any attempt to *somehow* merge these frameworks would erase the respective advantages.

### 3.1. Critique

An enormous number of theories (see also below) have been developed between these diametrically opposing endpoints of a spectrum of theories. The present work aims at a principled call for theoretical clarity and precision of terminology. Although some examples will be mentioned, it does not represent a critique of individual, specific research.

Based on ecological psychology (44) and connecting it to DST, the terminology partly changed to “ecological dynamics” [e.g., (45–47)]. The inclusion of “dynamics” may create the impression that the theory in question is founded in DST with its deterministic explanation of system behaviour but the terms are used inconsistently. For example, Rudd et al. (48) considered the domains of environment, task, and individual and discussed the learning aspects *within* the perspective of the coach or teacher. The complexity of the learning process is referred to as “non-linear pedagogy” [ibid, pp. 4; cf. also (49)]. The use and combination of such terms, for example, together with “constraints” and “ecological dynamics,” may propel the idea that the theory is be grounded in DST. Wood et al. (50) considered the coach as the third domain of interest (next to environment and task). Others focused on the athlete–environment interaction within an “ecologically situated perspective” ((51), p. 18). Furthermore, the athlete–environment interaction should embed emotions in the interplay of cognitions, actions, and perceptions (52). The quoted studies illustrated the imprecise combination of terms, which may lead to individual interpretations and, potentially, misunderstandings. There is no doubt that in the above-quoted examples, the separate concepts (e.g., emotion, task, coach, and athlete) are important and exert critical influences in skill learning, but the concepts need to be differentiated, and terms should be used consistently.

Regarding psychology, Gelfand and Engelhart (53; cf. also 17, 19) pointed out a potential confusion of dynamic systems with static models. Critically, dynamic models state that the system state depends on the same or other variables at an earlier point in time. This time dependency must not be confused with “non-linearity” (53). By analogy, DST may describe similar patterns of system behaviour as seen in motor skill improvements (15), but the need to prove DST as the explanation (which requires identification of *all* relevant variables and *exact* measurements) cannot be satisfied [cf. (53, 54)].

### 3.2. Other frameworks

Many theories recognised that the body (next to a symbol-manipulating device) and the environment must be considered to understand human behaviour. Gibson (44) emphasised the role of environmental factors, calling the framework *ecological psychology*. Accordingly, the environment interacts with the athlete [e.g., (56, 57)] and must be considered.

Factors related to the body have been discussed in the *embodiment* framework [(57) for review]. Here, it is emphasised that cognition is *not* independent of the body (and the situation). That is, cognition is seen “to serve the needs of a body interacting with a real-world situation” (ibid., p. 635). Crucially, the connection between the mind and body is bi-directional. Sensorimotor representations are seen as the basis for mental simulation, which is at the core of mental training.

Similarly, the ideomotor framework proposes bi-directional links between perception and action [cf. (58, 59)]. A related theory is the perceptual–cognitive approach to voluntary movement coordination (60, 61). Interesting evidence and arguments are provided for the *leading* role of (rhythm) perception in executing movements. Although much work is devoted to cyclic and often bimanual actions, this theory has also been applied to skilled movements such as the tennis serve (62) and other sports (32). Mechsner (60, 61) suggests the existence of movement representations, highlighting that they are sparsely coded (no full body parameterisation) and that perception (including re-afferent body signals) has a leading role in motor control.

The environment, as well as the task, has an impact on our actions and skill learning (25, 63) [for an action-centred perspective, see (64, 65)]. The task represents the action goal, which is allocated on the psychological (symbolic) level. Importantly, the action is also embedded in the social situation. These insights have been incorporated conceptually within the idea of systems theory [e.g., (20, 66)] without a mathematical formulation as in DST. This is reasonable because systems theory emphasises synergies among (interacting) components, and from lower levels, novel functional properties can emerge at higher levels (e.g., mental states or social phenomena such as team cohesion). Since all factors (environment, person, and task) are considered in such a perspective, it might be conceived of as comprehensive and at least conceptually holistic.

### 3.3. Considering a wider perspective

Given the frameworks discussed above, one may consider further factors that impact motor performance. In addition to coaches providing direct input to athletes to support skill improvements (i.e., movement-specific instruction and feedback), one should consider factors that impact motor performance indirectly. Such indirect (i.e., movement-unspecific) influences may range from team building [e.g., (67)] and leadership [e.g., (68)] to coaching [mentoring; e.g., (69)] and counselling [career development; e.g., (70, 71)]. An indirect input can stabilise performance, and tools of mental control can address emotion regulation (i.e., coping) and motivational (e.g., goal setting and routine development) and volitional aspects [e.g., self-talk and imagery; cf. (32, 72)].

To understand (psychologically) the needs of an athlete, a qualitative method, that is, a phenomenological or hermeneutic approach, would be adequate [e.g., (73); see (74, 75) for reviews]. To support an athlete who is continuously evolving, the back-and-forth of exploration and understanding (i.e., hermeneutics) appears appropriate for indirect (i.e., movement-unspecific) support.

## 4. Conclusion

With its inherent deterministic properties, DST provides a mechanistic description of complex system behaviours *without* a dedicated control structure. In contrast, SPA is focussed on abstract representations and symbolic manipulations (including verbal input) in motor planning. Hence, these frameworks are incommensurable, each with their own advantages. More recent approaches do take the role of the body and the environment (both physical and social) into account, for example, embodiment, ecological psychology, and ideomotor framework. However, a combination of terminologies (of DST and SPA) may lead to confusing theoretical concepts and core assumptions (e.g., deterministic processes vs. openness to symbolic influences). A differentiation of terms, concepts, and frameworks is required for theoretical advancements. Regarding applied work, a wider perspective may include indirect support, that is, understanding the needs and the (whole) situation of the athletes. Indirect factors such as emotional support or motivational guidance are movement-unspecific but may be critical for (optimal) motor performance. Employing these factors requires a (qualitative) *psychological understanding* of the individual athletes and their personal situations. Thus, they may be construed as the cognitive future of motor control and learning.

## Data Availability

The original contributions presented in the study are included in the article/Supplementary Material, further inquiries can be directed to the corresponding author.
